# Transactions of the Medical Society of the State of New York, for the Year 1858

**Published:** 1858-10

**Authors:** 


### ART. III.—*Transactions of the Medical Society of the State of New York, for the Year* 1858.

In many respects the volume of the Transactions of the New York State Medical Society for the present year, presents a favorable and agreeable contrast with the volumes of previous years. Its general typographical appearance is good, and the semi-white paper which has, under Legislative sanction been so long paid for by the people of the State of New York, at the price of a first rate article, is replaced by an article of unsullied purity. Moreover, the six hundred and fifty-five pages of this volume, are clothed in a decent garb; the poor apology for a covering which has heretofore been confined to the "fig leaf" of yellow paper covers, has given place to the full dress of a neat and tasty cloth binding, so that it is fit at once for full companionship with the most select of the company upon one's library shelves.

We much regret, however, that the sense of general satisfaction which is felt when one first takes the volume in hand, and turns over its pages, is sadly marred and gives place to poignant disappointment upon perusal, in encountering the almost innumerable and certainly gross typographical inaccuracies and blunders which deface nearly every page of the volume. The page of "Errata" at the end of the volume, does not compass a tithe of the errors of the book. Bad spelling, wrong words, misplaced and transposed sentences and paragraphs, meet you upon every side.

There has yearly been grounds for just such complaints. The State Society owes it to itself and the profession of the State, to devise some means by which its transactions shall not be perpetually sullied by these errors of the printer, and the negligence of the proof readers. If the other volumes emanating yearly under the sanction and seal of our legislature are as full of errors, we would give but little for their legal, historical, or scientific accuracy, or worth.

In commenting thus upon the verbal inaccuracies of the volume, we do not wish to be understood that the value of the book is to be judged alone by its mechanical execution. Far from it. Its contents have an intrinsic value of their own. But to meet these errors in the perusal, jars upon the nerves of the reader, as do discords in music upon the ears of the musician. Besides, these errors are not always free from the danger of lamentable results. For instance, take the prescription upon page 281, in Dr. Brinsmade's paper, where *one drachm* of the alcoholic ext. nux vomica, is substituted for *one scruple*, and *an ounce* of aloes for from *one* to *two drachms*. A corrected formula of the above, distributed we presume, by Dr. Brinsmade, we accidentally saw, and we presume we shall do the profession an acceptable service in aiding to disseminate the correct recipe:

℞ Alcoholic ext. nux vomica, 1℈.

Aloes (best quality), ʒj to ʒij.

Mix and divide into sixty pills.

There are other errors in the prescriptions given in the volume, occasioned by substituting wrong signs of quantities.

To turn from these mechanical blemishes to the consideration of the contents of the volume, is a task more within the appropriate sphere of the reviewer, and where there is so much to commend, a more agreeable duty. The contributions consist of twenty-six distinct articles, and furnish a large range in the subjects communicated. Twelve plates are added as illustrations to the contents of the papers.

Many of the articles scarcely admit of criticism; some are biographical sketches of departed members of the profession; others are the report of cases and make no other pretension than the legitimate one of recording medical or surgical phenomena, keeping clear of the regions of theory, or fancy.

The volume opens with the *Annual Address* of the President, Dr. Augustus Willard, upon *Air, Exercise and Sunlight*, a well written, earnest paper, the object of which is to exhibit the influences exerted over human health by the free use and enjoyment of a plenty of pure air and sunlight, conjoined with exercise, and the suffering occasioned by their want. He regards these as exerting as important influences upon human health and life, as any of the causes which develop diseases known under the classification of zymotics. A disregard of the laws of nature imposed upon us for the preservation and security of health, gives rise as frequently to diseases of a chronic character, whose progress towards a fatal termination is just as certain and just as inevitable as from causes commonly regarded as epidemic, and that the results of one series of causes is as truly fatal and terrible in their consequences as the other; with this advantage upon the side of epidemics, that there is a chance for escape from an attack of even the most fearful of them, but from many of the diseases engendered from a want of pure air, sunlight, and exercise, the escape is certainly hopeless after they have once become fixed in the system; sooner or later the foundations of life are undermined, and the doomed victim falls a sacrifice.

The three succeeding papers contain biographical memoirs of the late Prof. Thomas Spencer, and Drs. Sumner Ely, of Otsego county, and Henry Reynolds, of Saratoga. They are grateful tributes to the memories of faithful men in the profession, who have performed well their duties in the warfare of life and died with their armor on. It is fitting thus to gather the memorials of the worthy dead of our profession, and thus to place upon record the history of their devotion to the cause of humanity and of science, and to erect to their memories the monuments which the public, in whose service their lives have been spent, will never erect.

*Anæsthetics* furnish the theme for two papers. The first by Dr. Peter Van Buren, of New York, a general consideration of the subject of *anæsthesia;* the second by Dr. John G. Orton, of Binghamton, *on the use of amylene as an anæsthetic.*

Dr. Van Buren's paper is a species of report, or resumé of the employment of the various forms of anæsthetic agents in medicine and surgery. He enters into a recitation of the historical order in which these several articles were introduced, and what is of full as much importance, succinctly gives their chemical properties, and characteristics. Chloroform as matter of course, from the prevalence of its use, receives the greatest amount of consideration, and seems to be taken as a general type of the anæsthetic agents, and is employed almost as a synonym for the whole class. The new agent, *amylene*, receives an appropriate notice.

The various uses to which these agents have been put, are all respectively considered, and the paper furnishes a convenient means of reference to the practitioner upon all the points involved in the consideration of their application in medicine, obstetrics, or surgery. The only portion of the paper which we can spare space, or time to specially notice, is that which refers to the purity of chloroform. He says:

"Much of the unpopularity attaching to the use of chloroform in this country, is owing, doubtless, to its impurity. Until quite recently, very little of foreign preparation, of any description, has been imported, and of a pure article a still more limited supply, and that by a few members of the profession, for their individual use merely. The profession, generally, have rested satisfied with the article supplied by our druggists, without much inquiry or solicitude about its quality. The preparations in use have been manufactured chiefly in Philadelphia, and by an examination recently instituted, have been ascertained to be quite impure. That made by Powers & Weightman, which is the best, and which has been more generally used than any other, is still far from pure, containing, as has been demonstrated by tests to which it has been subjected, ingredients deleterious to health. The article supplied by Delue, a French preparation, and which is the only foreign chloroform that has been used to any extent, until within a few months, although considerably below the standard of purity, is yet superior to that made by Powers & Weightman. There is a third preparation in use, also made in Philadelphia, by Kuribaum & Co., and which is worse than either of the others."

Three preparations, at a late period, have been introduced to the notice of the profession, and which are represented to be pure: That made by Dr. Squibb, of Brooklyn, N. Y., at the Naval Laboratory, and expressly for the use of the U. S. Navy, and is not an article of commerce; from Cross & Young, also of Brooklyn, N. Y., and which is very little, if any, inferior to the former; and the well-known preparation of Duncan, Flockheart & Co., of Edinburgh. It is only within the past year that this has been imported to any considerable extent. It has been regarded by those who have used it, as one of the best, if not the very best preparation of chloroform manufactured.

Upon the means of testing the purity of chloroform we quote the following:

"The acid test, by which the quality of chloroform can be most readily determined, was first recommended by Mr. Morson, of London, in 1848, and more recently, agained urged upon the notice of the profession, by Dr. Squibb, U. S. N.

"Take equal quantities of pure sulphuric acid and chloroform, and mix them in a test-glass, or small bottle, with ground glass stopper, and having shaken the mixture, set aside for a few minutes to separate. If the chloroform is pure, no change will be perceived in the appearance of the acid, or temperature of the mixture. If, on the other hand, it be impure, it will at once be seen in the color imparted to the acid. The degree of impurity will be plainly disclosed by this test—the slightly impure causing a light brown, and the still more inferior preparations, a dark and almost inky appearance. This experiment will also, in some degree, determine its strength; since if water is mixed with it, the increase of temperature, upon the addition of the acid, will be perceptible to the touch."

Dr. Orton's paper is brief, and contains the results of his own observations and experience in the use of *amylene* as an anæsthetic. He was able to obtain none of the effects attributed to this article by the English surgeons, from any specimen he could obtain here in the shops, or which he could manufacture himself. But a specimen obtained from the laboratory of Bullock & Reynolds, of London, manifested all the anæsthetic properties described by the English surgeons.

Dr. Orton has been exceedingly pleased with its effects, and regards it as more rapid in producing anæsthesia than either chloroform or ether; a smaller quantity is required; the recovery from the state of anæsthesia, is almost instantaneous; and its administration is accompanied by fewer unpleasant symptoms.

The three contributions upon *Cerebro-Spinal Meningitis*, by Drs. D. G Thomas, of Utica, Oneida county; J. V. Kendall, of Clay, Onondaga county; and T. H. Squire, of Elmira, Chemung county, are among the most important of the volume, being descriptive of a new, and terribly fatal epidemic, which suddenly developed itself in the central and southern portions of this State, during the winter of 1857, and for the fatality accompanying it, and the suddenness of its termination, the cholera scarcely furnishes a parallel, some of the cases terminating in two or three hours from the time of seizure.* The date of the outbreak of the epidemic seems fixed in the month of February, 1857, although Dr. Squire reports a case occurring as early as the 23d of December, 1856, and another on the 1st of January, 1857; and Dr. Kendall reports a fatal case on the 8th of the same month; but the cases accumulated so rapidly and were so marked by their peculiar characteristics in February, that their diagnosis was no longer involved in doubt, and the terrors of a severe and fatal epidemic encompassed the inhabitants of these several localities, or more properly, districts.

Dr. Thomas' paper is more strictly confined to the description of the symptoms, and the pathological conditions accompanying them than the other two, he does not enter into any description of individual cases.

From his historic account of the advent of this disease in an epidemic form, we learn:

"It appeared in various garrisons in France, in 1837, and continued until 1842. In 1838 it prevailed in Lamollé and Leipsic; in 1839 in Versailles, Avignon and Strasbourg. It was epidemic in Gibraltar in 1844, and was not confined to the garrison, but prevailed extensively among the civil population. In 1846–7, it prevailed in England and Ireland. In March, 1848, it was epidemic in Alabama, Missouri, and Arkansas. About the same time it suddenly broke out in Sutton and Millbury, Massachusetts; and in several localities in central New York, in 1850–51. It made its appearance in Elmira, N. Y., during the warm weather of February, 1857, and prevailed during the two succeeding months extensively in the western and central parts of the State.

"Wherever it has prevailed in an epidemic form, it has been evident that an agent of uncommon power was sapping the fountains of life. Its usual ratio of mortality has been from seventy to eighty per cent. It is true that in some places the ratio has been less than seventy, while in other localities the mortality has been more than eighty per cent. The first nineteen cases treated in Sutton, Massachusetts, all died; thirteen cases in the French garrison, at Doesia, all died; and in the epidemic in Boon county, Missouri, five-sixths died."

---

* Reference may also be appropriately made in this connection, to the article by Dr. J. W. Craig, of Churchville, N. Y., in the Buffalo Medical Journal for July last, page 65.

Dr. Squire says:

"It occurred in Sydenham's day, and he describes it as typhus petechialis novus. It occurred in many parts of New England, in 1807 and 1808, at which times Dr. Elisha North, of Hartford, published an interesting monograph upon the subject, under the title of Spotted Fever. Many of the physicians now residing in Cortland and the adjoining counties of this State, will remember an epidemic which prevailed there about ten years ago, and was generally called cerebro spinal-meningitis."

Our contributors agree in ascribing to the disease a malarious origin, and in evidence upon this point, Dr. Thomas quotes authorities. Without stopping to quote his foreign proof, we copy only his statements that

"Its ravages continued in Alabama during wet weather, when there was an unusual degree of moisture in the atmosphere, the wind most of this time in the south-west. In the spring of 1844 the Missouri river overflowed a large section in the county of Boon, depositing a layer of sand over a large growth of vegetable matter. During the next three years this section, covered with the deposit, was unusually healthy, while beyond its limits it was sickly. In 1848 this disease appeared with fearful malignancy, but confined entirely to the country covered with the deposit from the river. In Alabama two hundred and forty cases occurred in low moist localities, to ten on the slopes and tops of hills. It broke out in Elmira during the warm weather of February. The place has no sewerage, and the surface water and filth was exposed to an unusual high temperature for the season. It has almost invariably prevailed during the damp weather of March and April, or a warm February, but few cases occurring after the first of May, and appearing again in the month of November and a warm wet December."

There is a very marked uniformity in the description of the symptoms detailed by these several contributors. Dr. Kendall, however, describes with the greatest care, the successive steps by which the disease makes its inroad upon the system, and the minute differences of symptoms as developed in individual cases, and relates seven cases of the disease. In the effort to condense our notice into a reasonable brevity, we shall, however, make use of the more synoptical description of Dr. Thomas, and as he has evidently employed the force of condensation to his description, we shall quote his language entire:

"Its mode of attack is not uniform. In some cases, its approach is accompanied by some disturbance of the stomach and bowels, as slight nausea and vomiting, and moderate diarrhœa. This state is accompanied with slight chills and pain in the head and back, sometimes very severe. After a short time, perhaps a few hours, these slight disturbances of the system will be followed by delirium, an anxious countenance, great restlessness, cool or cold skin, and a frequent *irregular* pulse. As the disease progresses, their muscles become affected with spasmodic contractions, and especially those of the back and neck, which often assume a permanent rigidity, confining the body or neck to one position for days, and sometime even weeks. The above is one of its modes of attack; but, in the strongly marked congestive form, the irregular pulse, the cold surface, and coma more or less profound usher in the disease. In the course of a few hours, reäction becomes established, the pulse becomes frequent and strong, and not unfrequently retains its irregular action; the skin hot and dry, urgent thirst, severe pain in the head and back, local and general spasms, torpid bowels, irritable stomach ejecting green morbid secretions, delirium, head drawn to one side, or fixed firmly backward, *petechiæ*, and an irritable and excessively tender surface. The morbid condition of the tongue varies with the progress of the disease; in the early stages being slightly covered with a white, or yellow brown fur, and later in the disease, dry and red, or a dark brown coat is found to cover it."

Dr. Squire has detailed the symptoms and termination of forty-three cases, and contents himself with the recitation of the symptoms and peculiarities of each individual case, without entering upon a more systematic description of the disease.

The petechiæ accompanying the disease, or developed in its course, form a marked feature among the other symptoms, and are worthy of a separate notice. Dr. Kendall says:

"Sometimes as early as the second day, but later, if not a very severe case, petechial spots appear, first upon the face, neck and breast, and extending more or less over the whole surface. These spots are very various in their appearance. In size they are from a mere point to three or six lines in diameter, and even larger in some instances. In color, from a scarlet to a dark purple—I had almost said black. In frequency, from a few scattered spots to one-fourth of the whole cutaneous surface. Where they are few and scattered, they are more generally of a scarlet or rose-red color; when more abundant and larger, of a darker hue. These remain upon the surface but a few days if the patient live, gradually fading, and in a week they are all gone. If the patient continue to sink, the petechiæ continue, and continually grow darker till death.

"Probably a majority have no petechiæ at all, at least not discernible by the naked eye; even some of a fatal character have not exhibited them."

The term "spotted fever" has been applied by some to the disease from the accompanying petechiæ.

"The post-mortem examinations," according to Dr. Thomas, "in all the localities where it has prevailed, show great similarity. The serous membranes of the spinal cord and brain are more or less congested, and effusion of serum, of coagulated lymph, and if the inflammation has had time to pass through its different stages, sero-purulent matter and pus—a layer of plastic purulent matter covering the whole inner surface of the pia mater of the brain, and collections of the same about the base of the brain, the pons nasali, and the medulla oblongata. Beneath the spinal arachnoid is found the same kind of purulent matter, and often pus is found opposite the third and last dorsal vertebræ. The spinal marrow, cerebellum and cerebum, have sometimes been found softened, but they are not usually affected; when they are, the disease assumes a remittent or an intermittent form."

There is nothing peculiar in the modes of treatment adopted. They were such as were generally indicated by the type and symptoms of the disease, and may be generally described as counter-irritants, and supporting means. Bleeding was illy borne, except in a few exceptional cases. Calomel was beneficial, but the chief reliances were opium, quinine, counter-irritation to spine, cold to head, and supporting the powers of life.

*The Report on the Comparative use of Ergot and the Forceps, in Labor*, by Dr. B. Fordyce Barker, is a concise statement of the relative value of these two obstetrical aids in retarded labor, and the points are so succinctly stated, and follow each other in such regular order, that we should be compelled to give the report nearly, if not quite, entire, were we to attempt to follow it. He first speaks of the action of ergot, and its use and abuse in labor, and then of its value for other purposes, connected with parturition; and passes to the use of the forceps in labor.

We extract the following from his third paragraph upon the use of ergot in labor, "The exhibition of ergot is safe only in those cases, where the presentation is natural, the pelvis is well formed, the os uteri well dilated, the vagina and vulva lax and moist, and in short *every thing is prepared for delivery, nothing being wanted but efficient action of the uterus*." The dangers from its use, he gives as rupture of the uterus, laceration of the os uteri, rupture of the perineum, also prolapsus and procedentia of the uterus and bladder. Injurious effects upon the mother's system are at times attributed to its use; and fatal effects upon the child when its delivery is not accomplished within a limited period of time, are results now established beyond all controversy.

He evidently favors the use of the forceps as a means of delivery in retarded labor, and regards the dangers from their employment as the results of their abuse, or ignorance in the mode of applying them. He thinks laceration of the perineum more frequently results from sloughing caused by pressure of the impacted fœtal head than from rupture from the employment of the forceps.

The paper is disfigured by numerous faults of the type setter. We may instance one, where what should be the first line of page 118 is inserted as its last line; and again, on page 127, "within *one year*," is put down as one of the periods of the duration of labor! Nor are these all the reader will find.

*Osseous Union of Intracapsular Fracture of the Neck of the Femur*, is a paper contributed by Prof. Alden March, in which he advocates "the doctrine of complete fracture within the capsular ligament, and union by ossific deposite, without impaction." He gives a case which he substantiates by the testimony of eye witnesses of the accident, or persons conversant with the history of the patient. The patient, a negro, was about sixty years of age, at the time of his death, the accident occurring when he was twelve or fourteen years old. An illustrative plate accompanies the article.

Certain we are that a shaft (*of bone?*) in the report is aimed at a professor of surgery in our midst, of no mean reputation, and who is building up for himself an enduring monument of fractured and dislocated bones. The reference, we think, to the "American Professor of Surgery," might have been more gracefully made, without detracting from the merits of the report.

Prof. March also contributes an *Interesting Case of Urinary Calculi*, occurring in a lady aged eighty-seven. She voided upon one day, after suffering from pain in the lower part of the body, fifteen calculi. The symptoms continuing, five and a-half months afterwards, Dr. M. discovered a calculus in the bladder, which he seized in the jaws of a small sized dressing forceps, and endeavored to deliver through the urethra, but upon traction, the calculus gave way and the instrument was detached. By introducing the forefinger of his right hand into the vagina, and pressing upon the bladder behind the location of the calculus, and introducing the broad or scooped extremity of a common pocket case director, he succeeded with the finger and instrument, in bringing the calculus into the urethra endwise. Maintaining it in this position, with a straight probe-pointed bistoury introduced flatwise on the upper and outer face of the calculus about an inch and a-half, he cut upwards and outwards towards the groin. The anterior extremity of the urethra was divided from a third to half an inch, which was amply sufficient to enable him to bring the calculus forward, and to extract it by the aid of the finger and grooved staff. The patient speedily recovered her health perfectly.

Prof. March mentions incidentally in his contribution, that he has operated for lithotomy twenty-one times, for lithotripsy three times. His youngest patient was two years and ten months old, his oldest the subject of this report. His oldest male patient was seventy-two years of age.

He makes a fitting notice, as a conclusion to his paper, of Prof. Benjamin W. Dudley, of Lexington, Kentucky, now over seventy years old, who has operated for lithotomy two hundred and seven times, and with a success unparalleled in the annals of capital surgical operations, having lost but five or six patients out of this large number.

Dr. Franklin B. Hough, in a brief paper *On the Registration of Births, Marriages and Deaths*, urges the importance of collecting these statistics, from their value to science, and their important bearing upon numerous questions of the highest interest in the social economy. Questions of sanitary and quarantine regulations are not alone among the problems to be wrought out by such statistics, but questions in life insurance and other financial affairs, dependent upon the contingencies of life; the determination of the numerous legal points connected with consanguinity, descent and inheritance; the application of mathematics to the solution of the great problems of vital statistics, are to be solved by the data thus obtained, and these subjects ought to be only named to enlist the sympathy and claim the attention and support of every member of the community.

*Poisoning by Arsenic from Absorption*, is contributed by Dr. C. V. Barnett, of Windham Centre, and is the recitation of twenty-one cases, several of which he details at length, where unequivocal signs of poisoning resulted from the external applications of this mineral, the work of a so-called "cancer doctor;" the application made use of was in every case a mixture of arsenic and nitric acid, applied liberally with a swab, and all, with two exceptions, occurred within the short period of one year.

The long continued use of the hyd. per-oxide of iron, with anodynes and mucilaginous drinks, was the treatment employed, and with the most marked benefit. He regards the doctrine of the books, that such cases are to be treated upon "general inflammatory principles," and that "blood-letting is our principal remedy," as unsound and unsafe.

An interesting case of *Accidental Nigrities*, occurring in a female at the age of sixteen years, next follows, from Dr. W. H. Gardiner, of Whitesboro.

The paper entitled *Human Longevity*, inserted on page 245, omitted in the numbering of the series of papers, but which finds a place twice in the table of contents (for some unexplained cause,) appearing at last as Art. 21, must have been inserted as a matter of personal compliment to its author. For although the address is profusely ornamented with *italics* and SMALL CAPS and *quotations* of poetry, and is the annual address of its president, we cannot discover the points of interest about it, which led the Albany County Medical Society "to communicate" it to the State Society for publication in its Transactions. If there are any, we should be most happy to have them pointed out. If it is designed to establish this as a precedent, there are doubtless other county societies in the State, which will desire to compliment their presidents in a similar manner. Such a rule we cannot but think would be as often honored in the breach as in the observance, judging by the present example.

Although we have noticed so many distinct articles, and omitted some others, for our space will scarcely permit us to give a synopsis of each paper, we have only so far indicated the contributions which furnish comparatively a small portion of the entire volume. *Two hundred and ninety-nine* pages of the six hundred and fifty comprising the volume, are the contributions of Dr. Thomas C. Brinsmade, under the title of the *Vice-President's Address.* At the previous meeting of the society, by resolution, it was made the duty of the Vice-President hereafter to deliver an address at the annual session of the society. While complying with the requisition of the society, he avails himself of the opportunity afforded, to present a series of elaborate tables, being a record of his practice for a period of twenty-one years. The cases are arranged and classified according to the nosology proposed by Dr. Wm. Farr, of England, and are extended until they cover two hundred and fifty pages! fourteen pages more are occupied with a "Statistical Nosology."

These tables are preceded by the *Address.* Referring to the action had from time to time in the American Medical Association, and in the New York State Medical Society, relative to the collection and preservation of medical statistics, he adopts the subject as the theme of his discourse, and urges with all the earnestness of which he is master, the great importance to medicine as a science of the daily record by physicians of their cases, and making the results of their observation and experience available to the profession. The enthusiasm which he himself feels upon the subject, and the importance which he attaches to the results to grow from such records, can, perhaps, be no better exemplified than by a reference to the immense amount of time and labor which he must have devoted to the preparation of these tables. Few men would have had the nerve to undertake such a work, extending back for so great a length of time, and but very few in the State, we opine, have any record of their practice for twenty-one years which could be worked up into any such form.

In the contemplation of such labors, prompted by an undoubted love for his profession, and stimulated by a noble enthusiasm to contribute to its advancement, it is an ungracious task to say aught which may be construed to their disparagement, or which may seem to detract from their value.

Notwithstanding the sympathies we naturally have for all statistic gatherers, for we know full well from our little experience, of the tread-mill character of the toil involved, and how few realize the tedium of the task which is imposed upon him who attempts to collate such facts as are presented in the tables of Dr. Brinsmade, we feel constrained to inquire into the practical utility of so many figures.

What we shall have to say, we promise will be less in disparagement of the labor of Dr. B. than for the purpose of instituting the inquiry as to the real utility of these laborious records when carried out to the full extent which they cover in the tables before us.

The profession for years has been lectured upon the importance and absolute necessity even, of collecting statistics for the purpose of developing the laws concerned in the production and propagation of disease, and the final termination in health, or death. The State Medical Society has committed itself fully to the task of endeavoring to obtain such statistics, and to work out the problems involved. A system of State medical registration has been proposed, and the results are waited for by those interested especially, with no little interest. It may not be unprofitable to inquire into the probable value of some, if not all, of the figures collected by such a widely diffused system of registration, which contemplates that every regular physician in the State shall keep a daily record of all the cases, medical and surgical, which he meets in practice.

In his enthusiasm, upon this subject, Dr. B. says:

"According to the census of this State for 1855, there are more than six thousand physicians in the State, and if but one in six of them would register, as proposed, we should have one thousand doing something every day, to a greater or less degree valuable. If they saw but four new cases a-day, four thousand facts would be daily placed in a tangible shape, ready for future use."

Well! suppose that one thousand medical men were heartily enlisted in this cause in the State, and that each at the next meeting of the State Society should present, as has Dr. B., his three hundred pages of printed statistics, what would we have?—why, *three hundred thousand pages*, or *five hundred volumes* of the same size as the present volume of Transactions! Truly the profession would have occasion to exclaim, "in the making of books there is no end," and the revenues of the State would groan beneath the taxation. We admit that this is a result no ways to be anticipated, but, while protesting against any desire to turn the subject into ridicule, we could not refrain from the opportunity thus afforded of showing how really impracticable would be the results of these record-takings if his anticipations or his desires could be once carried into effect.

We have stated that the State Medical Society has committed itself to this system of the individual registration of cases; its committee has prepared and sent out, designing to reach every member of the profession of the State, blank forms to be filled during the year, and to be returned in time to arrange and present at the next annual session of the Society. The plan is the carrying out of the suggestions contained in Dr. Brinsmade's address.

It is proposed that every physician shall daily register every case coming under his eye: not only all diseases, including epidemics, endemics, constitutional and local affections, but everything surgical, including accidents the most severe as well as the most trivial, from the dislocation or fracture of a femur, to the getting of a pepper-corn or bean into the nose, or a splinter in the toe. Look at Dr. B.'s paper and tables and see if we misstate the range of subjects.

Registration is the popular topic of the day; it is the theme of every convention; and the profession seems expectantly waiting for the revelation of some new and great truths from amid the chaos of recorded facts, to lead it out from the fogs and darkness which envelop it upon so many sides, into the brightness of a perfect day. The subject is a popular one, and not the least so with those who will never make a note, or attempt to arrange a table.

Notwithstanding the popularity of the subject, and the vast results expected to be achieved by this general system of registration, it may not be unprofitable upon the threshold of the enterprise to pause and inquire, whether there is any probability of all these grand results ensuing from this heterogeneous collection of heterogeneous facts.

We desire to ask these questions:—What good results are likely to flow from this collection of figures involving every conceivable form of lesion of function, or of organ, medical or surgical? What laws are to be deduced from the collection of a list of accidents, many of them of the most trifling and puerile character which can come under the eye of the medical man?

There is no difference of opinion as to the knowledge to be learned from the record and comparison of cases of disease which spring from epidemic or endemic causes, or such as impress the system from influences from without; but what laws are to be deduced from the register of cases of diseases constitutional in their character, and which spring from causes inherent within the patients themselves? What laws are to be deduced from the simple record of the fact of disease, when the record stands simply as the statement of a fact, isolated from any of the concomitant circumstances upon which the disease may be supposed to be dependent, or owe its origin? Again, what laws are to be deduced from the record of simple and pure accidents? Is there any connection between the months of the year and the frequency of the fracture of a clavicle, or the dislocation of a shoulder, or a thigh? Is a child more likely to swallow a button, or get a bean in its nose, or a pin in its ear at one season of the year than another?

That we do not seek to depreciate the value of this form of statistics, by stating suppositious records, we refer to the tables of Dr. Brinsmade, and which we take as our text in these remarks, where he has recorded all these cases, and elaborated them through all the tables comprising his twenty-one years record.

Will such daily records if preserved and published by the profession of the State, or any considerable portion of them, give us a true approximation to the real uumber of cases of disease? In acute diseases, running rapidly to a termination, they may, and most probably will. But how will it be with chronic and incurable diseases? It is not probable than any considerable number of persons suffer any length of time from any form of disease of a chronic, or incurable character without consulting more than one, or even two medical men. It is notorious that such cases "run the rounds" of the doctors of the neighborhood, and when their skill is exhausted go abroad for advice to whomsoever they may deem capable of aiding them in their extremities. If every one to whom they apply for advice record them among their daily observations, and return them in their yearly reports, you have a number of cases whose aggregate is formed by multiplying the patients by the number of doctors consulted. If the case terminate fatally, you have the record of one death only, against your swollen list of cases, and how are you to account for the balance? are they to be put down as cured, and ascribed to the advance of medical science?

Let us quote, by way of illustration, some of the cases of constitutional disease, with their recorded terminations, from Dr. B.'s tables, (pages 511 and 530.) In twenty-one years, he had 164 cases of dropsy, 18 fatal; cancer, 25 cases, 4 fatal; schirrus, 8 cases, 1 fatal; cancer of uterus, 6, *none* fatal; cancer of stomach, 7, fatal 4. But we have quoted sufficient. Now what is the inference from the records? that these most fatal forms of disease all recovered? The true explanation undoubtedly is, that the well-known reputation of the reporter brought these cases within his notice during some period of the disease, but that long before their termination they had passed from beneath his observation. Was he the only physician consulted by these patients during the period of their hopeless sufferings? We doubt it much. Suppose they were seen but by three others, a small number, and all recorded in their daily record of cases, and you have four cases to every actual patient affected. The disproportion to the actual reality destroys at once all value of the records. The mortuary list alone would furnish a true state of the case. We have selected these forms of diseases simply from their marked characters, but any one need but cast his eye over the long list of chronic diseases, to discover how many are contained therein, in which the same causes would operate to destroy the reliability of any tables.

We have intimated that the knowledge of the laws of disease deducible from such records as are here contemplated, has reference almost entirely to such as impress the system from influences from without, and that we shall derive little aid in our study of constitutional diseases.

In proof of this we can have no better evidence than Dr. Brinsmade's own paper. Turn to the really interesting and valuable charts, or tables which precede the address, and you will find but two classes of diseases as put down as being controlled by atmospheric, thermal, or terrestial causes—the zymotici and pneumonici. And as farther evidence upon this point, let any one carefully and studiously turn over the numerous tables of the book, and note the recurrence of cases month after month, and year by year, as they follow each other in succession, and mark the entire independence of the diseases not included in these two classes, from any relation to time or seasons. The only possible points upon which information can be gained, is in reference to the influence of age and sex in the development of disease, and upon many of these points our knowledge is now a certainty, but at any rate, to learn these facts is not necessary, or desirable, to be embarrassed by a vast amount of superfluous matter, although dignified by the name of statistics.

We can scarcely refrain the remark, that these tables of Dr. Brinsmade are invaluable to the profession, in exhibiting how really limited the field of observation is, when you come to measure it in this manner by rule and line.

We are fully impressed with the conviction that some rational bounds and limits must be placed to this passion for statistic gathering which now pervades the profession. It is not sufficient to add figure upon figure, and to accumulate table upon table, to work out the problems of health and disease, of life and death. An intelligent discrimination must be made in the facts recorded, and those only presented upon which you can predicate some reasonable grounds for supposing that the relation of cause and effect exists. To simply record that you have visited "four new cases" of disease to-day, teaches nothing. If you visit four more to-morrow, teaches nothing. You may be very busy, and your neighbor very idle; your families may be accidentally all sick at a time; his, all well. And if the visits are to some such cases as these tables exhibit, they serve no possible purpose or use,—except to advertise the extent of your business!

With the full consciousness of the fact, that medical-statistic-gathering is the popular hobby of the day, and that the expression of an opinion of doubt only, of its utility, is heterodox, we reiterate our conviction, that some rational bounds and limits must be marked out within which these labors must be confined, or else the whole subject will become a stench in the nostrils of the profession, and be banished as a nuisance beyond the pale of legitimate medicine.

The Report of the Committee of the King's County Medical Society on "*The Statutes of the State of New York, regulating the Practice of Physic and Surgery; the Rights, Duties and Immunities of Physicians; and their relation to the Medical Societies of the Counties in which they reside*, we commend to the careful perusal and attention of every physician of the State, who claims to be regular; and to those without the pale of a County Medical Society, it has especial claims upon their attention.

In passing, we may remark, that the legislature of this State, for 1857, stultified itself beyond any of its previous efforts in *leveling down* the guards previously placed around the public as protection against quackery, by enacting at the heel of the session a law authorizing the formation of homœopathic societies, and basing the powers thus conferred upon the act of 1813 in reference to the organization of medical societies. How two bodies can exist in the State with equal powers upon such matters, we will leave for our courts to decide, premising, however, that we presume no greater contempt exist in reference to the decision of our courts among medical men, than among lawyers.

But we have extended our notice of the Transactions far beyond the limits we first designed, and exceeded the space legitimately belonging to us, and we must bring our remarks to a close. There are several other papers which we have not indicated by their titles, interesting in themselves, and adding to the value of the volume; the greater portion of them, however, call for no especial notice. We think we have sufficiently indicated the range of subjects embraced in the volume.

It should be a subject of congratulation with the profession, that there is such an apparent evidence of vitality in the State Society as is betokened by the volume before us. It is evident that the profession is at work despite the want of appreciation upon the part of the public, of the value of these continuous labors in its behalf, and the chilling, blighting influence of the support given to quackery in every form and degree. It is some consolation, however, that in its hour of greatest need, the public turns to the regular profession for deliverance from the evils which beset it, and redemption from prospective ills. To whom does it look for the working out of the laws concerned in the development of epidemics, or the production of ordinary diseases,—to the regular practitioners, or to the innumerable horde of irregulars who overrun the land?

We have felt constrained, however, from our desire to behold our profession at the summit of professional eminence, to question the value of some of the contributions to the present volume. And had it not been from a desire to avoid the charge of being hypercritical, we could have extended our criticisms to other points, and other papers. We would much rather commend than condemn, and it is ungenerous by an excess of criticism to repress any aspirations towards a laudable ambition to contribute to the advancement of our science.

We cannot, however, in conclusion, withhold the expression, that we think that there is a conviction, and a growing one too, in the minds of the profession of the State, that there is a tendency in our brethren of Albany and immediate vicinity to centralize the advantages of the society about themselves, and to convert what influence it exerts to their individual and collective glory, forgetful of the great mass of the profession outside of the capital of the State, composed of living, active men, fighting manfully the battles of the profession, without the prestige of the glory reflected from the State House.

